# The Research Society

**Published:** 1918-08

**Authors:** 


### THE RESEARCH SOCIETY

In his presidential address before the American Medical Association in 1896, Dr. Nicholas Senn said : " Medical Societies are the great post-graduate schools for physicians; and medical journals their best text-books. "

The truth of the first part of this statement is apparent to all who attend medical meetings, but particularly so to those who have had the privilege of attending the meetings of the Research Society of the American Red Cross, organized to aid the American Expeditionary Forces in France. Since it takes a year, or perhaps two, to write the average text-book, and to get it into print, physicians can learn the technic for new operations, the modifications of old ones, the recent improvement in medical procedures, and the advanced methods in sanitation, only from current medical literature.

The thousands of physicians who are with the American Expeditionary Forces in France have found themselves, as military surgeons, in a foreign country, where most of them do not understand the language. They cannot attend meetings of medical societies to learn from the experience of others how to deal with the problems which are new to them, and they have no books and medical journals to which they can refer for advice. Therefore the need has been very great for instruction or training in the problems that have arisen during the war, many of which have been solved by our medical confreres in the French and British Armies.

To meet this need, last October the American Red Cross in France organized the Research Society to cooperate in, and to supplement, the instruction given to medical officers in the Medical Department of the American Expeditionary Forces.

Meetings have been held every month with an average attendance of about two hundred medical officers, consisting of heads of departments and representatives from the various hospitals and divisions of the American Expeditionary Forces. The subjects dealt with have been those of greatest importance to the surgeons of our fighting forces, who have thus been able to profit by the experiences of French and British authorities on various medical problems of the war. The presentation of these problems by some of the greatest living surgeons and medical authorities has proved to be of inestimable value to those who were privileged to attend these meetings. While representatives of the French and British Armies have taken the larger part in these discussions, many distinguished American physicians and surgeons have presented the American viewpoint.

The representatives of the various hospitals and divisions who have been present have reported the meetings at conferences with the medical officers connected with their units; and the heads of the divisions have carried into effect the practical and helpful suggestions that have been made by their confreres.

The benefits derived from the meetings of the Research Society have been far reaching. They have resulted in a closer acquaintance, and a more cordial relationship with our French and British confreres, which will be helpful not only during the war, but also during the peace that is to follow the crushing of the Hun's military power; for it will make for a broader understanding among the medical men of the three great nations that will continue to be allied in the interest of science and true culture, as well as in that of human liberty.

